# Early-Onset Hemophagocytic Lymphohistiocytosis and Inflammatory Neurotoxicity Prior to Post-transplant Cyclophosphamide: A Report of Two Cases

**DOI:** 10.7759/cureus.112660

**Published:** 2026-07-14

**Authors:** Kashish Shah, Brian C Betts, Maureen Ross, Alicia Lieberman, Marco L Davila, Shernan Holtan

**Affiliations:** 1 Internal Medicine, B.J. Medical College/Civil Hospital, Ahmedabad, IND; 2 Transplant and Cell Therapy, Roswell Park Comprehensive Cancer Center, Buffalo, USA

**Keywords:** allogeneic hematopoietic cell transplantation, cytokine release syndrome, hemophagocytic lymphohistiocytosis, immune effector cell-associated neurotoxicity syndrome, post-transplant cyclophosphamide

## Abstract

We report two distinct, early hyperinflammatory toxicities after human leukocyte antigen-mismatched allogeneic hematopoietic cell transplantation with post-transplant cyclophosphamide in adult recipients. Case 1 developed severe cytokine release syndrome with shock, respiratory failure, renal failure, hyperferritinemia (22,771 ng/mL), hypertriglyceridemia (1,024 mg/dL), and multiorgan dysfunction, consistent with secondary hemophagocytic lymphohistiocytosis; he improved rapidly after receiving emapalumab. Case 2 developed fever and abrupt encephalopathy on day +2 with negative infectious and neurologic evaluation and rapidly improved after tocilizumab, consistent with cytokine-mediated neurotoxicity resembling immune effector cell-associated neurotoxicity syndrome. These cases highlight diagnostic overlap with infection and the need for early immunosuppressive intervention.

## Introduction

In the post-transplant cyclophosphamide (PTCy)-based allogeneic hematopoietic cell transplantation (alloHCT) platform, donor stem cells and mature immune cells are given without immunosuppression, allowing for an inflammatory cascade to begin prior to the administration of PTCy on days +3 and +4. During this window of time without immunosuppression, patients may develop a cytokine release syndrome (CRS)-like inflammatory response, manifesting with fevers and sometimes hypotension and hypoxia. This pre-PTCy hyperinflammatory state is thought to be driven by donor T cell activation, similar to T cell-mediated CRS observed in autologous chimeric antigen receptor (CAR) T cell therapy [1]. However, more severe inflammatory toxicities classically associated with CAR T-cell therapy, including hemophagocytic lymphohistiocytosis (HLH) and immune effector cell-associated neurotoxicity syndrome (ICANS), are less well described after alloHCT. We report two illustrative cases that highlight the importance of early recognition and prompt immunosuppressive therapy to mitigate potentially life-threatening hyperinflammation. Such cases are important to report, as there are currently no validated diagnostic criteria or well-established treatment paradigms for these clinical scenarios.

## Case presentation

Case 1

A man in his late-50s with myelodysplastic syndrome/myeloproliferative neoplasm with fibrosis (del(5q), KMT2A mutation) underwent a 7/10 human leukocyte antigen (HLA)-mismatched unrelated donor alloHCT with thiotepa/busulfan/fludarabine conditioning and dose-reduced PTCy (35 mg/kg) for graft-versus-host disease prophylaxis. His history included prior human immunodeficiency virus infection with undetectable viral load on combination bictegravir, emtricitabine, and tenofovir, coronary artery disease, hypertension, stage 3 chronic kidney disease, and type 2 diabetes. On day +1, he developed fever (39.1°C), fatigue, and tachycardia (grade 1 CRS), with persistent high fevers over the next 48 hours followed by acute kidney injury, metabolic acidosis, oliguria, hyponatremia, hyperbilirubinemia, and progression to hypoxic respiratory failure requiring bilevel positive airway pressure (grade 4 CRS). Infectious workup (blood and urine cultures, Clostridioides difficile testing) was negative. He also developed a type 2 non-ST elevation myocardial infarction requiring nitroglycerin infusion.

**Figure 1:**
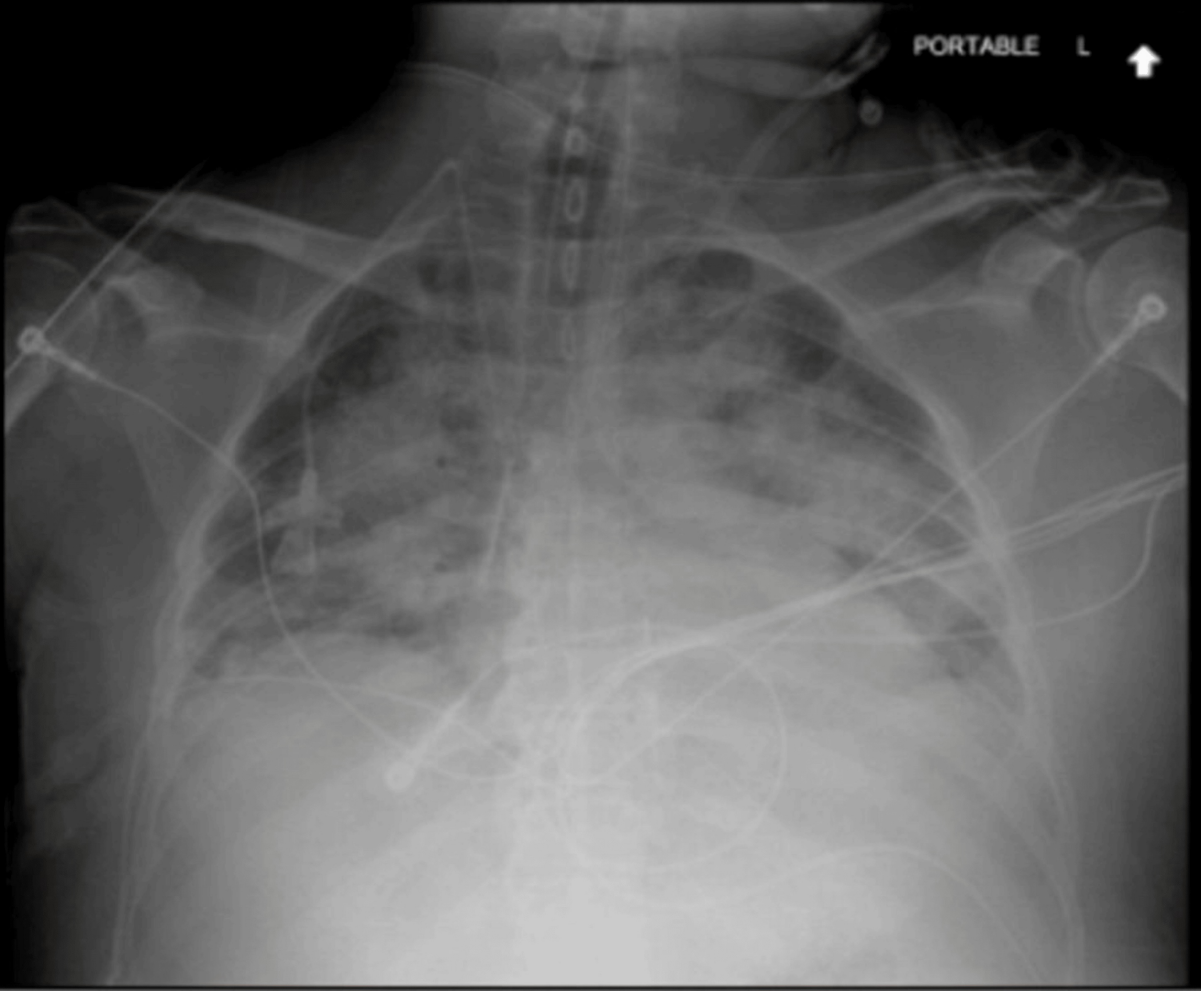
Chest X-ray showing extensive bilateral pulmonary infiltrates.

He received PTCy on days +3 and +4, but tacrolimus was initially withheld because of renal failure. On day +6, he received tocilizumab for unrelenting fevers suggestive of CRS, but he clinically deteriorated. Chest imaging showed extensive bilateral pulmonary infiltrates (Figure 1). He subsequently worsened with shock requiring vasopressors, acute renal failure requiring continuous renal replacement therapy, and intubation for respiratory failure. Extensive infectious disease evaluation was negative. Fever, hyperferritinemia, and hypertriglyceridemia supported a diagnosis of HLH. Ferritin was 22,771 ng/mL (reference range: 10 - 322 ng/ml) and triglycerides were 1,024 mg/dL (reference range: 44-200 mg/dl). He was pancytopenic from conditioning chemotherapy; thus, cytopenias could not help establish the diagnosis. Methylprednisolone 100 mg daily and ruxolitinib 5 mg twice daily were started on day +6. He was proned on day +7. A single dose of emapalumab 50 mg on day +7 led to improvement. Over the next week, he was weaned off immunosuppression, vasopressors, and ventilatory support, discontinued renal replacement therapy, restarted tacrolimus as renal function recovered, extubated on day +14, engrafted around day +21, and was discharged from the hospital on day +30 in stable condition.

In this case, fever, hyperferritinemia, hypertriglyceridemia, and multiorgan dysfunction supported a presumptive diagnosis of secondary HLH. Confirmatory testing, including bone marrow examination for hemophagocytosis, soluble IL-2 receptor, NK-cell functional activity, and prospective HScore calculation, was not obtained given the patient's critical illness, the limited sensitivity of marrow hemophagocytosis early in the disease course, and send-out turnaround times that would not have informed acute management decisions. While bone marrow examination for hemophagocytosis is part of the HLH diagnostic criteria, its sensitivity is limited early in the disease course, and send-out testing would not have altered the acute management trajectory. In this context, the combination of clinical features and rapid response to targeted therapy is sufficient to guide decision-making. 

Case 2

A woman in her late-50s with T-cell lymphoblastic lymphoma with an FGFR1 rearrangement underwent a 7/8 HLA-mismatched unrelated donor alloHCT using fludarabine, cyclophosphamide, and total body irradiation conditioning. Her past medical history included migraines, anxiety, major depressive disorder, neuropathy, cervical radiculopathy, overactive bladder, and adrenal insufficiency. On post-transplant day +2, she developed a high-grade fever (40.5°C) that did not respond to acetaminophen. By the following morning on day +3, she became progressively somnolent and obtunded, unable to follow commands and responsive only to sternal rub, without tremors or abnormal movements. Her vital signs remained stable, her head computed tomography was unremarkable, and she was transferred to the intensive care unit for close monitoring.

She started empirically on vancomycin, meropenem, micafungin, and acyclovir. On neurological examination, she was difficult to arouse, grimaced with noxious stimulations, pupils were sluggish but reactive, with conjugate gaze and blinking to threat, without tremors or clonus. Electroencephalography showed no seizure activity but diffuse slowing, and an extensive infectious evaluation (blood and urine cultures, respiratory viral panel, and serum viral and fungal studies) was unrevealing. Tocilizumab 800 mg was administered on day +3 due to concern for potential cytokine-mediated neurotoxicity, and her mental status improved dramatically within 24 hours, returning to baseline alertness and orientation. She received her post-transplant cyclophosphamide on days +3 and +4 as scheduled. Antimicrobials were eventually de-escalated. She subsequently engrafted and was discharged from the hospital in stable condition. She remains free of disease >1-year post-transplant with no long-term neurologic sequelae.

Her presentation was clinically suggestive of cytokine-mediated neurotoxicity resembling ICANS; retrospectively, this would have been consistent with American Society of Transplant and Cellular Therapy ICANS grade 3, and confirmatory advanced neurologic evaluation, including lumbar puncture, brain magnetic resonance imaging, and continuous electroencephalography, was deferred in light of her rapid clinical recovery following tocilizumab. Nonetheless, we advise caution; therapeutic response alone should not be interpreted as confirmatory evidence.

**Figure 2:**
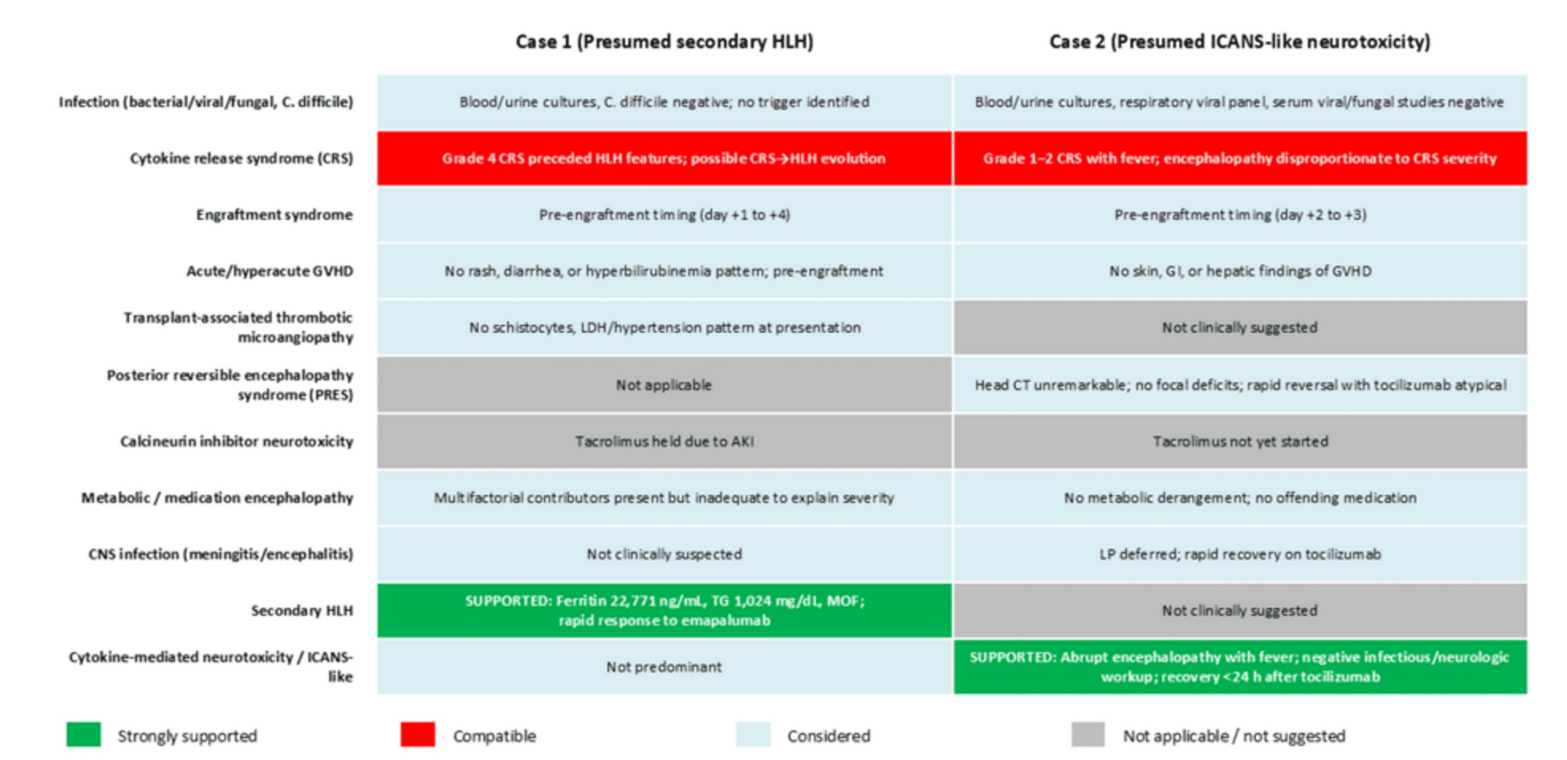
Differential diagnosis matrix for two cases of early hyperinflammatory toxicity following post-transplant cyclophosphamide (PTCy)-based allogeneic hematopoietic cell transplantation. AKI, acute kidney injury; CNS, central nervous system; CRS, cytokine release syndrome; GI, gastrointestinal; GVHD, graft-versus-host disease; HLH, hemophagocytic lymphohistiocytosis; ICANS, immune effector cell-associated neurotoxicity syndrome; LP, lumbar puncture; MOF, multiorgan failure; PTCy, post-transplant cyclophosphamide; TG, triglycerides. Each row represents a candidate diagnosis evaluated against clinical, laboratory, and temporal features for both cases. Color coding indicates the strength of diagnostic support: green denotes the strongly supported (favored) diagnosis, red denotes a compatible contributing process, light blue denotes alternatives that were considered but not favored, and gray denotes diagnoses deemed not applicable or not clinically suggested.

Differential diagnosis matrix for two cases of early hyperinflammatory toxicity following PTCy-based alloHCT is shown in Figure 2.

Side-by-side differential diagnostic assessment was performed for two patients who developed early post-transplant hyperinflammatory syndromes after PTCy-based allogeneic HCT. Case 1 presented with features most consistent with secondary HLH, supported by markedly elevated ferritin (22,771 ng/mL), hypertriglyceridemia (1,024 mg/dL), multiorgan failure, and rapid clinical response to emapalumab. Case 2 presented with abrupt encephalopathy and fever in the setting of a negative infectious and neurologic workup, with recovery within 24 hours of tocilizumab administration, most consistent with cytokine-mediated, ICANS-like neurotoxicity. CRS was compatible in both cases but insufficient to fully account for the clinical severity, raising the possibility of CRS-to-HLH evolution in Case 1 and a cytokine-mediated neurotoxicity phenotype in Case 2.

## Discussion

HLH is a life-threatening hyperinflammatory syndrome characterized by persistent fever, cytopenias, hyperferritinemia, coagulopathy, and progressive multiorgan dysfunction; it may be familial or secondary to an underlying trigger [2]. HLH is likely underrecognized and may increase as alloHCT use expands [3]. Post-transplant HLH may occur early (≤30 days) or late (>30 days) [4], and early cases are especially difficult to diagnose because they overlap clinically with infection, acute or hyperacute graft-versus-host disease, engraftment syndrome, CRS, and thrombotic microangiopathy [4,5]. In our patient, no infectious trigger was identified, and the initial presentation was attributed to grade 4 CRS, raising the possibility that severe CRS triggered or evolved into HLH, which is biologically plausible given shared cytokine-driven immune dysregulation [6]. Diagnosis is further complicated because hemophagocytosis may be absent early and cytopenias are common after alloHCT, making hyperferritinemia a particularly useful clue in this setting [3,7].

Reported risk factors for post-transplant HLH include haploidentical or mismatched transplant, peripheral blood stem cell grafts, high CD34 cell dose, reduced-intensity conditioning, and T-cell-replete grafts [8]. There is no standardized treatment approach for post-transplant HLH, but management generally requires rapid escalation of immunosuppression to control hyperinflammation [3,9]. Agents with emerging evidence include emapalumab (interferon-gamma blockade), ruxolitinib (Janus kinase inhibition), and tocilizumab (interleukin-6 blockade) [10-12]. In our patient, tocilizumab was given initially for presumed severe CRS, followed by corticosteroids and ruxolitinib, which also served as graft-versus-host disease prophylaxis while tacrolimus was held for acute kidney injury; emapalumab was added because of disease severity, with rapid clinical improvement.

Neurologic toxicity after alloHCT is described but nonspecific, with reported incidence ranging from 3% to 44% and a broad differential diagnosis that includes infection, metabolic derangements, medication toxicity, cerebrovascular events, posterior reversible encephalopathy syndrome, cytokine-mediated inflammation, and transplant-associated thrombotic microangiopathy [13,14]. Clinical manifestations range from headache and neuropathy to encephalopathy, seizures, and coma [15]. In our second patient, the early onset of altered mental status, temporal association with fever/CRS-like inflammation, rapid improvement after tocilizumab, and negative infectious and radiographic evaluation support an ICANS-like neurotoxicity syndrome similar to what is described after CAR T-cell therapy.

Our report has important limitations. Both diagnoses are clinical and presumptive, with differential diagnoses considered (see the reasoning matrix developed for these cases in Figure 2).

In Case 1, we did not obtain bone marrow examination to document hemophagocytosis, soluble IL-2 receptor levels, NK-cell functional testing, or a prospectively calculated HScore; these studies were not feasible in the time frame required for therapeutic decision-making. In Case 2, prospective ICANS grading was not applied, and advanced neurologic evaluation including lumbar puncture, magnetic resonance imaging, and continuous electroencephalography was not performed given prompt neurologic recovery after tocilizumab. We therefore frame both cases as clinically compatible with, rather than definitively diagnostic of, secondary HLH and ICANS-like neurotoxicity. These limitations underscore the broader point of our report: validated, transplant-specific diagnostic frameworks for hyperinflammatory toxicities after PTCy-based alloHCT remain an unmet need. CRS post-alloHCT, it will be recognized [16], but these more severe and rare inflammatory manifestations require further characterization.

## Conclusions

HLH and inflammation-associated neurotoxicity remain underrecognized yet potentially life-threatening complications of alloHCT. Diagnosis is particularly challenging in the early post-transplant period, as the clinical features, including fever, cytopenias, hyperferritinemia, end-organ dysfunction, and encephalopathy, overlap substantially with infection and organ toxicity, while pathognomonic findings such as hemophagocytosis on marrow examination may be absent. This diagnostic ambiguity, compounded by the absence of validated post-transplant diagnostic criteria, can delay recognition at precisely the point when intervention is most likely to alter the trajectory of disease. A high index of suspicion, serial monitoring of ferritin, triglycerides, fibrinogen, and a low threshold to initiate empiric immunosuppression in the right clinical context are essential. Although these syndromes have historically been linked to CAR T-cell therapy, they are being increasingly reported after alloHCT, particularly with HLA-mismatched grafts utilizing PTCy, in which a donor T cell-driven hyperinflammatory window precedes the immunomodulatory effect of cyclophosphamide on days +3 and +4. Diagnostic reasoning, as we demonstrate with these cases, may need to be applied in the future until larger case series can provide alignment on the clinical approach. As mismatched and haploidentical transplantation with PTCy continues to expand access to alloHCT, clinicians should anticipate this pre-PTCy inflammatory phase, recognize its potential to evolve into HLH or ICANS-like neurotoxicity, and be prepared to escalate promptly to agents such as tocilizumab, corticosteroids, ruxolitinib, and emapalumab. Prospective studies and consensus diagnostic frameworks tailored to the alloHCT setting are needed to standardize recognition and management of these rare but emerging toxicities.
